# The Positive Impact of the Early-Feeding of a Plant-Based Diet on Its Future Acceptance and Utilisation in Rainbow Trout

**DOI:** 10.1371/journal.pone.0083162

**Published:** 2013-12-27

**Authors:** Inge Geurden, Peter Borchert, Mukundh N. Balasubramanian, Johan W. Schrama, Mathilde Dupont-Nivet, Edwige Quillet, Sadasivam J. Kaushik, Stéphane Panserat, Françoise Médale

**Affiliations:** 1 INRA, UR1067 NUMEA Nutrition, Métabolisme et Aquaculture, Aquapôle INRA, Saint Pée-sur-Nivelle, France; 2 Aquaculture and Fisheries Group, Wageningen Institute of Animal Sciences (WIAS), Wageningen University, Wageningen, The Netherlands; 3 INRA, UMR1313 GABI Génétique Animale et Biologie Intégrative, Jouy-en-Josas, France; The University of Plymouth, United Kingdom

## Abstract

Sustainable aquaculture, which entails proportional replacement of fish-based feed sources by plant-based ingredients, is impeded by the poor growth response frequently seen in fish fed high levels of plant ingredients. This study explores the potential to improve, by means of early nutritional exposure, the growth of fish fed plant-based feed. Rainbow trout swim-up fry were fed for 3 weeks either a plant-based diet (diet V, V-fish) or a diet containing fishmeal and fish oil as protein and fat source (diet M, M-fish). After this 3-wk nutritional history period, all V- or M-fish received diet M for a 7-month intermediate growth phase. Both groups were then challenged by feeding diet V for 25 days during which voluntary feed intake, growth, and nutrient utilisation were monitored (V-challenge). Three isogenic rainbow trout lines were used for evaluating possible family effects. The results of the V-challenge showed a 42% higher growth rate (P = 0.002) and 30% higher feed intake (P = 0.005) in fish of nutritional history V compared to M (averaged over the three families). Besides the effects on feed intake, V-fish utilized diet V more efficiently than M-fish, as reflected by the on average 18% higher feed efficiency (P = 0.003). We noted a significant family effect for the above parameters (P<0.001), but the nutritional history effect was consistent for all three families (no interaction effect, P>0.05). In summary, our study shows that an early short-term exposure of rainbow trout fry to a plant-based diet improves acceptance and utilization of the same diet when given at later life stages. This positive response is encouraging as a potential strategy to improve the use of plant-based feed in fish, of interest in the field of fish farming and animal nutrition in general. Future work needs to determine the persistency of this positive early feeding effect and the underlying mechanisms.

## Introduction

Sustainable feeding practices in intensive fish farming require further reductions in the use of dietary inputs from fisheries [1]. Yet, studies in search for alternatives to fishery-derived fishmeal and fish oil demonstrate high reductions in growth due to high inclusion levels of plant-protein in fish feed [2], [3] as clearly shown in rainbow trout [4]. Low palatability of terrestrial plant-protein sources is considered a major constraint. For instance, alkaloids found in legumes such as peas and lupins reduce feed intake (FI) in rainbow trout without visible signs of adaptation [5], [6]. Likewise, purified alcohol extracts (e.g. saponins) from soybean appear to be feeding deterrents [7]. Independent of their effect on FI, high levels of plant proteins have also been shown to depress the efficiency of feed utilization [4], [8], pointing towards a digestive or metabolic problem. Other studies with salmonids showed that specific plant proteins such as soybean meal may provoke morphological changes and inflammation of the distal intestine [9], [10]. In contrast, changes in the dietary lipid source only have a minor impact on feed utilization and FI in salmonid fish [11], despite their capacity to discriminate and express specific feed preferences when given a choice among different feed oils [12], [13].

Due to the overall poor understanding of the physiological reactions of fish to specific plant components, other strategies to expand the use of plant ingredients are being looked into. In this respect, selective breeding studies in rainbow trout demonstrate the large potential to exploit genetic variability for improving the growth of trout fed plant-based diets [14]–[17]. An alternative strategy to ‘adapt the fish to the new feed’, relatively under-explored in the field of fish nutrition, is by means of early nutritional intervention. In mammals, it is now well established that early nutrition may permanently alter the organism's physiology and metabolism. This phenomenon is believed to have evolved as a mechanism that allows the organism to fine-tune its physiology in an adaptive way to its early milieu [18]–[22]. The time frame in which the programming can occur is often confined to critical or sensitive periods early in life [20] such as during fetal [19] or early postnatal [23] nutrition. In fish, existing literature indicates that the early exposure to dietary factors such as high carbohydrate content [24] and changes in fatty acid profile [25], [26] can induce persistent metabolic adaptations, at least at the molecular level.

Early nutritional events may not only influence an organism's metabolism or physiology, but also the development of sensory and cognitive systems [27]. Early exposure to quinine and citric acid, two substances innately aversive to rats, has been shown to reduce aversion to these tastes in rat later in life [28], [29]. Similarly, early flavor experiences in humans have been found to program life-long flavor preferences [30]. In salmonids, the function of the chemosensory system involved in feeding arises early. After emergence from the substrate, young salmonids display a synchronized anatomical, physiological and behavioral development, vital for the transition from endogenous (yolk) to exogenous nutrition (usually 20–29 days post-hatch). Morphological evidence suggests that the olfactory system is functional as early as hatching [31]. Newly-hatched fry, which do not yet take food, already display nonspecific motor responses to olfactory stimuli [32]. The taste system arises later, but rapidly develops at the time of exogenous feeding with the spectrum of effective taste substances expanding with age [32]. To our knowledge, the possibility to orient later feed flavor acceptance by early life exposure to specific feeds remains unexplored in fish.

The present study explores the potential to improve the acceptance and/or the utilization of a feed rich in plant-ingredients in rainbow trout, by means of early exposure to the same plant-based feed during the first three weeks of exogenous feeding.

## Materials and Methods

### Ethics statement

The experiments were conducted following the Guidelines of the National Legislation on Animal Care of the French Ministry of Research (Décret 2001-464, May 29, 2001) and in accordance with the boundaries of EU legal frameworks, relating to the protection of animals used for scientific purposes (*i.e.* Directive 2010/63/EU). The author who performed animal experiments holds a personal license from the French Veterinary Services. The experiment was conducted at INRA NuMeA (UR1067) facilities, certified for animal services under the permit number A64.495.1 by the French veterinary services.

### Experimental diets

Diets were manufactured at the INRA facility of Donzacq (France) using a twinscrew extruder (Clextral). The ingredient and analysed composition of both diets is given in Table 1. Diet M contained fishmeal and fish oil as protein and lipid source, respectively. Diet V contained a blend of palmseed, rapeseed and linseed oil, rich in saturated, mono-unsaturated and n-3 poly-unsaturated fatty acids, respectively, as lipid source. In order to avoid exceeding anti-nutrient threshold levels, we used a blend of wheat gluten, extruded peas, corn gluten meal, soybean meal and white lupin as protein sources. Synthetic L-lysine, L-arginine, dicalciumphosphate and soy-lecithin were added to diet V to correct the deficiency in essential amino acids, phosphorous and phospholipid supply. A mineral and a vitamin premix were added to both diets. Both diets fulfilled the known nutrient requirements of rainbow trout [33].

**Table 1:** Formulation, approximate crude protein (CP) levels of ingredients and analysed composition of the experimental diets M (fishmeal and fish oil-based) and V (all fishmeal and fish oil replaced by plant protein and plant oil sources).

Ingredients (g 100 g^−1^ diet)	Diet M	Diet V
Fish oil	8,5	-
Plant oil blend*	-	10,3
Fishmeal LT (CP 70%)	63	-
White lupinseed meal (CP 40%)	-	5,8
Corn gluten meal (CP 62%)	-	17,4
Soybean meal (CP 46%)	-	21,5
Wheat gluten (CP 80%)	-	25,6
Whole wheat (CP 10%)	25,4	5,1
Extruded dehulled peas (CP 24%)	-	3,1
Soy-lecithin	-	2,0
L-Arginine	-	1,0
L-Lysine	-	1,5
CaHPO4.2H20 (18%P)	-	3,6
Mineral and vitamin premix**	3,0	3,0
*Analysed composition*		
Dry matter (DM, % diet)	93,3	92,4
Crude protein (% DM)	52,1	50,5
Crude fat (% DM)	17,9	17,0
Gross energy (kJ g^−1^DM)	22,3	22,3

Consisting of (% blend): rapeseed oil (50), palm oil (30), linseed oil (20).

INRA UPAE, 78352 Jouy en Josas, France.

### Biological material

Three isogenic heterozygous families (all individuals within a family share the same genotype) of rainbow trout (*Oncorhynchus mykiss*) were produced (C1-A22, C2-AB1 and C3-R23), expected to differ in their growth response to a plant-based feed (based on our own unpublished data). The three families were obtained by mating a single homozygous female line with males from three other homozygous lines [34]. The use of the same maternal line avoids effects associated with egg size and hatching time. Ova were collected from different females from the same line in order to produce a sufficient number of fish. The ova were carefully mixed and divided into three groups, each group being fertilized by gametes from one of the three male isogenic lines. Family differences are thus due to the genetic variability brought by the paternal lines.

### Nutritional history and further pre-challenge phase

Hatching and first-feeding (23 days following hatching) took place at the INRA Lées-Athas fish farm, France (flow-through spring water, 7°C). For the first 21 days of exogenous feeding, the swim-up fry received either diet V or diet M, which was carefully distributed by hand on an hourly basis (8 to 10 meals per day) to duplicate groups (60 fry per tank). Each group was fed (7 min/meal) in slight excess. This early feeding period is referred to as ‘nutritional history V or M’ and fish from the respective nutritional histories are termed ‘V- or M-fish’ (Figure S1). The use of three genotypes (isogenic families) and two nutritional histories gave the following six treatment groups, C1V, C1M, C2V, C2M, C3V and C3M. During the period in between this early nutritional history period and the challenge test with diet V (V-challenge, see Figure S1), all groups were fed with diet M (hand feeding, 2 meals per day until visual satiation). Intermediate growth and feed intake was followed by weighing the fish groups and amount of feed distributed on a three-week basis. Survival was monitored daily. For accelerating the juvenile growth phase, fish were reared from ∼2 g body weight at higher water temperature, 16.5°C (INRA Donzacq farm, flow-through spring water). Three weeks prior to the V-challenge, fish were transferred for acclimating to the INRA facilities of St. Pée-sur-Nivelle, consisting of a recirculating water unit of 24 tanks (70 L volume, 7 L/min water exchange rate, 16.5±1°C water temperature and artificial photoperiod set at 13 h light). The V-challenge was carried out with 4 replicate tanks (18 fish/tank) per treatment group and 3 replicate tanks (17 fish/tank) for treatments C1V and C3M for which a replicate tank was lost during acclimation (due to a blocked water inlet). Feed intake (FI) was recorded during the last 7 days of acclimation (diet M), once stabilized in all groups.

### V-challenge

The V-challenge took place 7 months after the early nutritional history period (Figure S1). Here, all six treatment groups received diet V for 25 days. Rearing conditions were the same as during acclimation. For monitoring voluntary FI, two meals per day were carefully distributed by hand (diet V). Morning feeding started at 7:40 am and afternoon feeding at 2:00 pm. Specific care was taken to feed the groups to ‘visual satiation’. Each tank was fed in three feeding rounds, the last until complete arrest of feeding activity. Fish were given approximately 15–20 min to recover appetite between each feeding round. The few pellets which remained unconsumed were counted and subtracted from the amount distributed, by multiplying their number with the mean pellet weight. We thus ensured that FI was recorded as precisely as possible.

For measuring initial (BWi) and final (BWf) body weight (BW), fish were counted and group-weighed at the start and end of the trial. Specific growth rate (SGR) was calculated as 100*(ln(BWi)−ln(BWf))/25 days. Daily FI parameters, based on the total amount of food consumed divided by the number of days, were expressed on an individual basis (g/ind.day) or corrected for differences in growth, *i.e.* per 100 g average body weight (% BW.day) or per kg average metabolic body weight (g/kg met BW per day). Average BW was calculated as (BWi+BWf)/2 and average metabolic BW as ((BWf/1000*BWi/1000)∧0.5)∧ 0.8. Feed efficiency (FE) was calculated as BW gain/total dry matter intake. For analysis of whole body composition, 6–8 fish (36-h unfed) per tank were sampled the day of initial and final weighing, killed by an overdosis of anaesthesia (phenoxyethanol, 0.5 ml/l), frozen and kept at −20°C prior to biochemical analyses. Ground feed and whole fish samples (freeze-dried) were analysed for dry matter (105°C for 24 h), ash (combustion in a muffle furnace, 550°C for 12 h), protein (acid digestion, N×6.25, Kjeldahl Nitrogen analyser 2000, Fison Instruments, Milano, Italy), lipid content (petroleum ether extraction, Soxtherm, Gerhardt, Germany) and gross energy (adiabatic bomb calorimetry, IKA, Heitersheim, Germany). The retention efficiency of protein, lipid and energy was calculated as 100*(BWf*X-BWi*X)/FI*Y, with X being the percentage protein or lipid or the amount of energy (kJ/g) in the fish and Y that in the feed.

### Restricted V-challenge with focus on feed utilization efficiency (FE)

A restricted V-challenge was performed in order to investigate the effect of nutritional history on FE, independent of possible confounding effects related to differences in FI. For this, the fish received during four weeks an identical amount of diet V. We applied a restricted daily feed ration which was set at 0.75 g of feed per 100 g BW. Observations during the V-challenge suggested this amount to be readily consumed by all groups. The amount of feed distributed was adjusted to the tank's biomass after two weeks, following an intermediate group-weighing. The ration was distributed by hand (2 meals/day) and special care was taken to ensure that all food distributed was consumed. The restricted V-challenge took place 13 months after the early diet V/M exposure. We used the remaining nutritional history M and V fish (only families C1 and C2 were available) from the same batch as in the V-challenge, kept at 7°C. As for the V-challenge, these had been fed with diet M from the end of early exposure until the first day of the restricted V-challenge. The four groups (C1M, C1V, C2M, C2V) were tested in duplicate tanks with 11 and 25 individuals per tank for the C1 and C2 treatments, respectively. The average BW of the fish at the start of the restricted V-challenge was not affected by nutritional history (P = 0.61) but was higher (P<0.05) in fish of family C2 than of family C1 (59.0 and 55.4 g, respectively). The restricted V-challenge was performed at the same temperature as the V-challenge (INRA Donzacq fish farm, flow-through spring water, 16.5°C).

### Statistical analyses

Statistical analyses were performed using STATISTICA 7.0 (StatSoft Inc., Tulsa, USA). Data were tested for normality and homogeneity of variances by Kolmogorov-Smirnov and Bartlett tests, and then submitted to a two-way ANOVA to test the significance of the effects of nutritional history (N Hist), family (Fam) and their interaction (FxNH). In case of a significant effect (P<0.05), means were compared by Newman-Keuls post-hoc test.

## Results

### Performances of the fish during the pre-challenge phase

**Figure 1:**
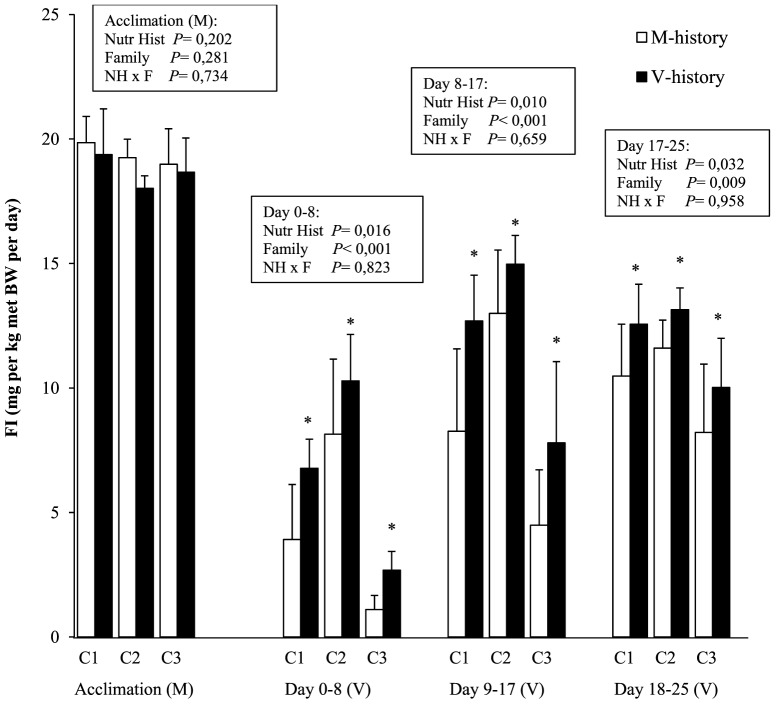
Voluntary feed intake (FI) of the trout during the last week of acclimation when feeding diet M and over three consecutive periods of the 25-day V-challenge with diet V. FI data represent means ± SEM (n = 4, except for C3M and C1V with n = 3) according nutritional history (M or V) and family (C1, C2, C3). For each period, the significance of the effects of nutritional history, family and their interaction (2-way ANOVA) is provided in the figure, * indicates a significant effect of nutritional history (V>M, p<0.05).

The percentage survival during the almost 8 month pre-challenge phase (including the nutritional history phase) was 89±6%. Survival was not significantly affected by nutritional history or family (P>0.05). The body weight of the fry at the end of the 3 weeks of first-feeding (nutritional history), was significantly (P<0.001) affected by nutritional history and by family: all M-fish were significantly bigger than V-fish. The early growth was highly dependent on the family in fish fed the V-, but not the M-diet, as shown by the statistical interaction between both factors (P<0.001), giving the following body weight ranking C3M, C2M and C1M (0.17 g)>C3V (0.12 g)>C2V (0.10 g)>C1V (0.08 g). The growth trajectory of the fish during the rest of the pre-challenge phase showed a similar pattern among all groups (Figure S2). At the end of the pre-challenge phase (start of the V-challenge), no effect of nutritional history was noted on the body composition (Table 2) nor on the body weight of the fish which ranged between 33.5 g and 42.1 g according to the family (Table 3). Fish dry matter content and protein level was family-dependent (C1 = C2>C3, Table 2). The average daily FI on diet M, measured at the end of the pre-challenge phase (last 7 days of acclimation), was unaffected by family or previous nutritional history (Figure 1).

**Table 2:** The body weight (BW) of the trout at the start of the V-challenge and body composition of the fish at the start and at the end of the V-challenge.

Family	C1		C2		C3			P value (2-way ANOVA)		
Nutritional History	M	V	M	V	M	V	SEM	Fam	N Hist	FxNH
*Body composition of the fish before the V-challenge*										
Dry matter (% BW)	28,1	28,1	27,4	27,2	25,9	26,3	0,27	0,002	0,783	0,582
Protein (% BW)	14,0	13,5	13,8	14,3	13,1	13,4	0,14	0,008	0,591	0,013
Lipid (% BW)	11,5	11,2	10,5	10,5	10,2	10,3	0,15	0,067	0,496	0,627
Energy (kJ g^−1^ BW)	7,3	6,8	7,1	6,8	6,4	6,3	0,13	0,440	0,107	0,666
*Body composition of the fish at the end of the V-challenge*										
Dry matter (% BW)	29,3	28,9	30,1	30,5	27,4	28,8	0,28	0,002	0,257	0,245
Protein (% BW)	14,5	14,3	14,8	14,8	14,1	14,7	0,09	0,022	0,611	0,926
Lipid (% BW)	12,3	12,9	13,1	14,0	10,4	11,9	0,27	0,000	0,002	0,931
Energy (kJ g^−1^ BW)	7,8	7,9	8,5	8,6	7,0	7,9	0,13	0,000	0,003	0,011

Data represent treatment means according to their early nutritional history (M or V) and family (C1, C2, C3). P-values (2-way ANOVA) show the significance of the effects of nutritional history (N Hist), family (Fam) and their interaction (FxNH).

**Table 3:** Data on growth, feed intake and nutrient utilization of the trout during the 25-day V-challenge.

Family	C1		C2		C3			P-value (2-way ANOVA)		
Nutritional history	M	V	M	V	M	V	SEM	Fam	N Hist	FxNH
**Growth parameters** (g ind^−1^)										
Initial body weight	33,5	33,8	36,1	34,6	38,7	42,1	0,70	0,000	0,103	0,001
Final body weight	46,6	57,0	61,3	65,2	45,3	55,9	1,8	0,000	0,001	0,299
Protein gain	2,07	3,59	4,08	4,72	1,32	2,60	0,29	0,000	0,003	0,510
Lipid gain	1,92	3,56	4,20	5,48	0,79	2,34	0,37	0,000	0,001	0,910
Energy gain	122	218	264	323	69,2	176	20,7	0,000	0,001	0,643
**Voluntary feed intake (FI)**										
FI (g ind^−1^)	15,4	23,0	24,9	29,2	9,5	15,9	1,6	0,000	0,005	0,760
FI (% BW d^−1^)	1,52	2,02	2,03	2,33	0,89	1,29	0,12	0,000	0,011	0,834
FI (mg kg BW^−0.8^ d^−1^)	8,1	11,2	11,5	13,3	4,7	7,1	0,7	0,000	0,005	0,769
**Nutrient and energy utilization efficiency** (% intake)										
Protein retention	28,4	33,5	35,1	34,6	29,3	35,1	0,8	0,069	0,017	0,117
Lipid retention	75,5	98,3	107,9	119,5	43,9	90,8	6,2	0,001	0,003	0,203
Energy retention	37,8	46,0	51,3	53,9	34,5	53,3	1,9	0,005	0,001	0,045

Data represent treatment means according to their early nutritional history (M or V) and family (C1, C2, C3). P-values (2-way ANOVA) show the significance of the effects of nutritional history (N Hist), family (Fam) and their interaction (FxNH).

### Growth performance, feed intake and feed efficiency during V-challenge

**Figure 2:**
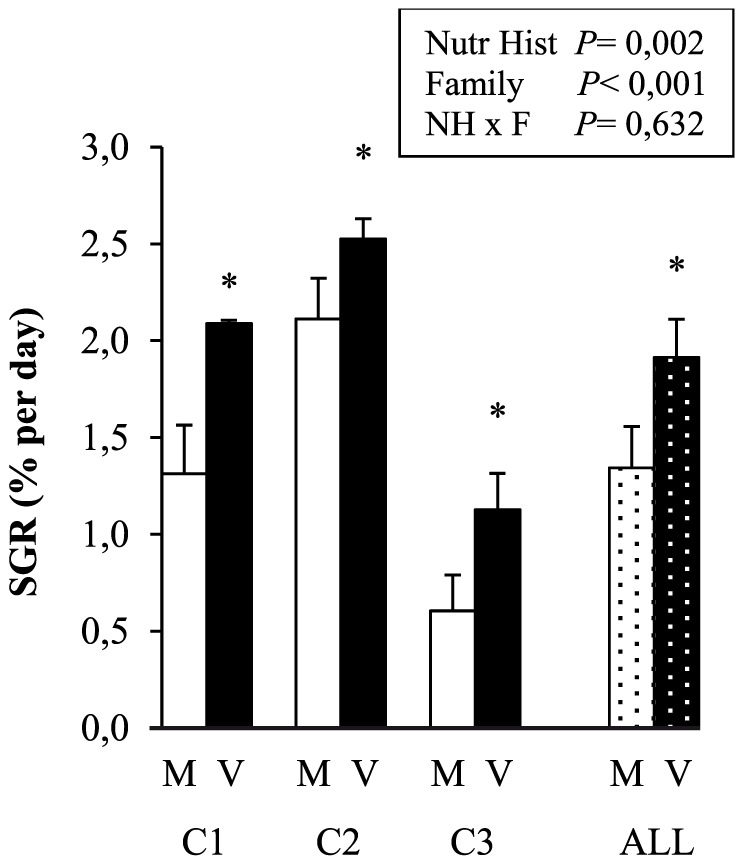
Specific growth rate (SGR) of the trout during the 25-day V-challenge according to the early nutritional history (M or V) and family (C1, C2, C3). Values are means ± SEM (n = 4, except for C3M and C1V with n = 3). Dotted bars represent the effect of nutritional history (M or V) during the V-challenge, averaged over all three families (ALL, means ± SEM, n = 11). The significance of the effects of nutritional history, family (C2>C1>C3) and their interaction (2-way ANOVA) is added in the figure, * indicates a significant effect of nutritional history (V>M, p<0.05).

The specific growth rate of the fish during the V-challenge (Figure 2) was on an average 42% higher in V- (1.9%/d) than in M-fish (1.3%/d). The whole body total lipid content was affected by nutritional history (V>M, Table 2). The major components of body weight gain, *i.e.* protein and lipid gain, were (averaged over the three families) 48 and 65% higher in V- compared to M-families, respectively (Table 3). There was a significant family effect on the latter parameters, C2>C1>C3, without family*nutritional history interaction.

Voluntary FI (g/fish), cumulated over the entire V-challenge (day 0–25), was 37% higher in V- compared to M-fish (Table 3). This difference was 27% and 30% when expressed per unit average body weight or per unit metabolic body weight, respectively (Table 3). FI differed between the three families, with family C2 consuming significantly more of diet V than C1 which in turn had higher intakes than C3 (C2>C1>C3). The absence of a significant family*nutritional history interaction shows that differences in FI due to nutritional history were independent of the family effect. *Ad libitum* FI data are detailed in Figure 1 for the three consecutive periods of the V-challenge, *i.e.* days 0–8, 9–17 and 18–25. Three major observations are noteworthy. First, the transition from diet M (and acclimation to diet V) resulted in a huge drop in FI in all groups, as seen during the first period (d0–8) of V-feeding. Secondly, in all periods and for all families (no interaction), V-fish consumed significantly more of diet V than M-fish. This positive effect of early V-exposure on FI was more marked during the first week (V/M ratio of 1.80) than during the last week (V/M ratio leveled off at 1.21). Thirdly, the family effect was significant in all 3 periods with highest FI in C2 groups (C2M, C2V) and lowest FI in C3 groups (C3M, C3V).

**Figure 3:**
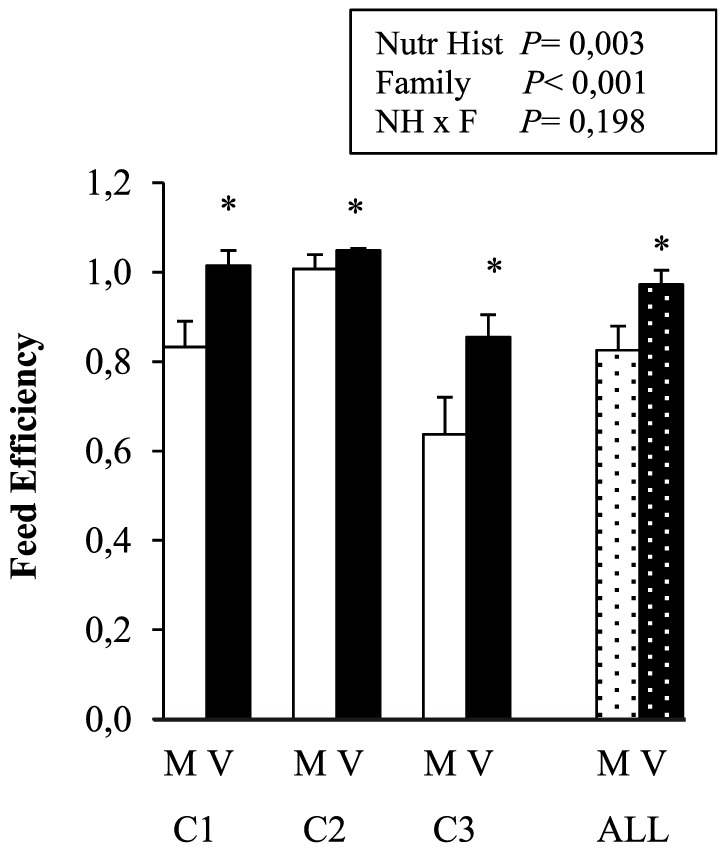
Feed efficiency (FE) of the trout during the 25-day V-challenge according to the early nutritional history (M or V) and family (C1, C2, C3). Values are means ± SEM (n = 4, except for C3M and C1V with n = 3). Dotted bars represent the effect of nutritional history (M or V) during the V-challenge, averaged over all three families (ALL, means ± SEM, n = 11). The significance of the effects of nutritional history, family (C2 = C1>C3) and their interaction (2-way ANOVA) is provided in the figure. * indicates a significant effect of nutritional history (V>M, p<0.05).

Feed efficiency (FE) was significantly affected by both nutritional history and family (Figure 3). With a FE of 0.97±0.11, V-families gained on average 18% more in weight per unit FI than M-families which had a mean FE of 0.84±0.18 (Figure 3). FE in fish of family C3 was lower than that in C1 which was lower than in C2. There was no significant interaction between both factors, though the positive effect of nutritional history V on FE seemed somewhat less pronounced in family C2. The efficiency of protein, lipid and energy retention (gain per unit intake) was, respectively, 11, 36 and 24% higher in V- compared to M-groups (Table 3).

### The efficiency of feed utilization during the restricted V-challenge

**Figure 4:**
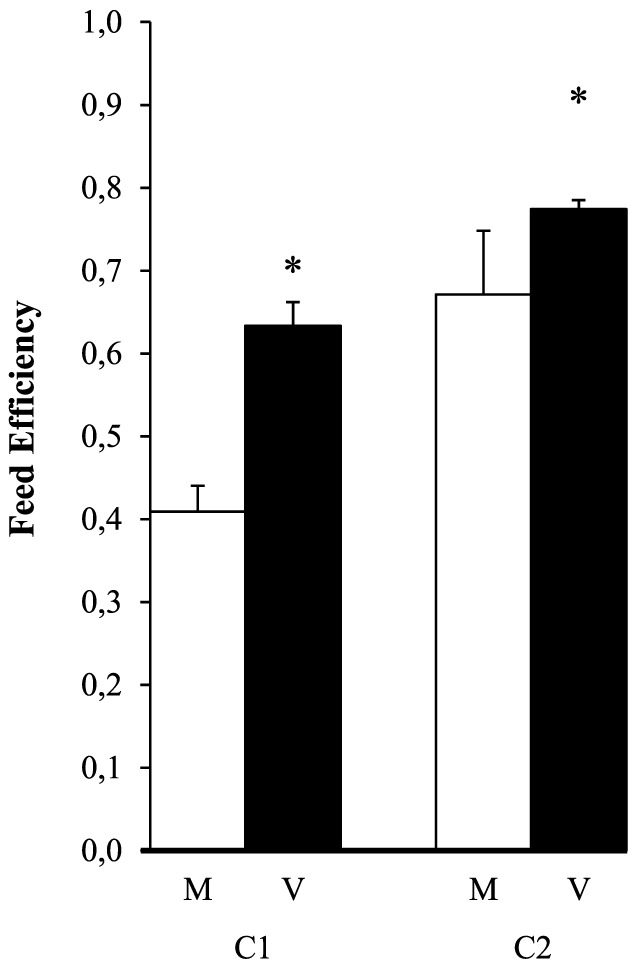
Feed efficiency (FE) during the restricted V-challenge. Two families of rainbow trout with nutritional history (M or V) received for 4 weeks diet V at 0.75% of their body weight (restricted feeding). Values are means ± SEM (n = 2). The significance of the effects of nutritional history, family (C2>C1) and interaction (P values, 2-way ANOVA) is provided in the results section, * shows a significant effect of nutritional history (V>M, p<0.05).

The 4-week restricted V-challenge confirmed the positive effect of early diet V-exposure (P = 0.02) and the effect of family (P = 0.01, C2>C1) on the utilization efficiency of diet V, without significant interaction between both factors (P = 0.24, Figure 4).

## Discussion

The juvenile rainbow trout that were confronted during early first-feeding stages with the plant-based diet V displayed better growth when fed this same diet 7 months later (V-challenge) compared to the non-exposed M-fish. The better growth in the V-fish is attributable to a combination of higher *ad libitum* FI together with better feed utilisation. To our knowledge, so far no study in fish has documented an analogous positive long-term effect of short-term early exposure to a plant-based diet.

### V-exposed fish display higher feed intakes (FI) during the V-challenge

FI drastically dropped in all groups challenged to eat diet V, devoid of both fishmeal and fish oil, as frequently seen in salmonids fed diets with high levels of plant ingredients [2]. Key to the present study is the finding that the drop in FI was significantly less prominent in V- than in M-trout which had never been confronted before with diet V. Cumulated over the V-challenge and depending on the family, V-exposed fish consumed 20 to 70% more than M-fish. This long-term positive effect needs emphasis, taking into account the over 300-fold increase in fish body weight between the V-challenge (35–40 g fish) and the early V-exposure (∼100 mg fry). Further research should assess the persistency of the observed effect as the contrast in FI caused by nutritional history steadily decreased over time.

In any event, the effect of early nutritional history on FI implies that the V-fish were able to ‘recognize’ diet V, and this 7 months following initial exposure. The capacity of flavour learning has been shown before in teleost fish, albeit over a shorter time span than in the present study and mostly by applying a conditioned aversion paradigm in which flavor is associated with a noxious stimulus. Goldfish has been found capable to learn to avoid flavored food particles following injections with lithium chloride for periods of 11 days [35] or 47 days [36] after learning. Both gustatory and olfactory systems seem to be involved in the initial learning process [36] whereas the dorsomedial telencephalic pallium seems to play an essential role in memorizing the associative food aversion in analogy with the amygdala in mammals [35]. Likewise, long-lasting aversive associations between the sensory properties of a food and its postingestive consequences have been repeatedly highlighted in generalist herbivores [37]. Such associative memory is considered crucial for aiding the animal to avoid particular plant-toxins during foraging [38], [39]. Importantly, the early V-exposure in our study did not result in an aversion to diet V as might be expected in case the trout had associated the early diet V experience to negative postingestive consequences. Instead, the juvenile fish early exposed to diet V ate more during the V-challenge compared to the M-fish. This is, in essence, strongly suggestive of reduced food neophobia, which may be mediated through mechanisms related with sensory flavor acceptance or with reduced susceptibility to specific plant secondary compounds (e.g. enhanced detoxifying capacity).

In mammals, early flavor experiences are important in establishing life-long food flavor acceptances [30] and may render distasteful flavors palatable [28], [29]. The ability to retain nutrient flavors transmitted by the mother's diet (amniotic fluid, milk) has been interpreted as a natural mechanism for the safe transmission of predictive dietary signatures from mother to young [40], [41]. In fish, knowledge on the effect of early flavor experience on later food flavor acceptance is scarce. In salmonids, olfaction is believed to be more important in guiding feeding behavior than gustation [42]. Moreover, gustatory preferences show low plasticity in fish and have been reported to be independent of previous feeding experience [32]. Of interest, though not directly related with the development of feed flavor acceptance, is the susceptibility of the salmonid olfactory system to imprinting, a mechanism used by adult salmon to find their way back to the natal streams [43]–[46]. The olfactory imprinting process is assumed to be linked to major physiological processes (e.g. emergence from the gravel, smolt-parr transformation) and external environmental clues (e.g. exposure to novel water) [47]. Olfactory imprinting under laboratory conditions has been shown to work with compounds such as morpholine or phenetyl alcohol [44], [48]. More recent studies indicate a role of free amino acids (L-isoforms), probably derived from a variety of living organisms (e.g. plants in and near streams), as guiding substance for salmonids to return to their natal river [46], [49], [50]. In our study, specific compounds released in the water from diet V during the trout's early-life exposure perhaps provoked an olfactory imprinting, responsible for the reduced neophobia and higher intakes later in life. The prospect of alleviating food flavor neophobia in fish by early short-term flavor exposure certainly warrants further attention.

Food neophobia is also considered as an innate reaction which prevents the animal to ingest potentially harmful unknown substances [38], [39]. In this respect, two mechanisms, not mutually exclusive, may underlie the higher FI seen in the V-trout later in life. This is i) the trout fry had learned during early V-feeding that no severe harmful substances were associated with diet V consumption or ii) the early V-exposure stimulated physiological defense mechanisms to deterrent plant substances. In terrestrial herbivores, early exposure to plant secondary compounds can permanently alter critical physiological detoxification systems [51]. Persistent modifications in xenobiotic metabolism by early plant-feeding have not been considered yet in trout or any other fish.

The amount of food eaten during the V-challenge differed significantly between the three isogenic families, as expected from previous results on paternal effects on *ad libitum* FI of plant-based feed using isogenic rainbow trout lines [52]. Such genetic variability in FI in trout fed plant-based diets is considered of particular interest for setting up selective breeding program [14]–[17], [52]. In humans, genetic differences in the sensitivity to taste substances interfere with early experiences in establishing food likes and dislikes [30]. In our study, however, the effect of early V-exposure on later FI appeared consistent for the three families (no statistical interaction).

### V-exposed fish display improved feed efficiency (FE) during the V-challenge

Besides FI, also the efficiency of the utilization of diet V was higher in V- relative to M-fish. Analysis of the components in body gain showed that this was associated with better retention efficiencies of both lipid and protein. The improved capacity of the V-fish to utilise diet V for growth appears promising, but requires caution regarding i) the possible confounding effect of FI on the observed FE-response and ii) the diet specificity of the FE-response.

Regarding the first point, FE in fish is known to show a positive quadratic relationship with feeding level [53]–[56]. Using good quality feed, optimal FE normally occurs at 20 to 25% below the maximum growth response level, whereas it rapidly declines at the lower intake ranges [56]. This relationship which is feed-dependent is often overlooked in nutritional studies where FI and FE are mostly interpreted as independent parameters. Our data do not allow to estimate the impact of the lower intakes in M- relative to V-groups on the observed reductions in FE. This was the reason for undertaking the small-scale feeding trial in which fish of both nutritional histories were fed restrictively at 0.75% of their body weight. The low feeding levels ensured all feed to be consumed but led to lower growth. Nevertheless, the data confirm the positive effect of early V-exposure on later FE seen during the V-challenge, in this case more than one year after the early exposure.

For the second question, it is important to say that actual FI during the early feed exposure could not be monitored due to the small size of the feed pellets (300–500 µm) and fry (<200 mg). It is hence conceivable that V-fry consumed less than M-fry during these first weeks of exogenous feeding, despite the hourly feed supply provided in slight excess to all groups. In mammals, early-life exposure to a nutrient-limited environment has been reported to lead to hyperphagia and obesity later in life, probably as a result of metabolic dysfunctions programmed by early nutritional deprivation [19]–[21], [57]–[60]. We therefore conceived the possibility that a general early ‘malnutrition’ effect, resulting in overcompensation, might explain the superior ad libitum FI and/or dietary utilization in the V-fish during the V-challenge. However, no such compensatory feeding effects were seen in the V-fish fed with diet M during the 7-month intermediate rearing, as also reflected by the similarity in fish body mass at the start of the challenge which was independent of nutritional history. This clearly points toward a directed diet V response in the juvenile V-fish rather than to an overall compensatory sign of early malnutrition in the V-fish.

A wide range of nutritional conditions and compounds has been found to induce specific adult phenotypes in terrestrial animals and man. When encountered during early life, these may provoke long-lasting adaptive changes in preparation to the potential future environment. If the predicted nutritional environment is correct then the organism's metabolism will match, increasing its evolutionary fitness [18]–[22]. The foresaid literature has particularly dealt with ‘mismatches’ as in the case of fetal undernutrition and with nutrient-induced programming of genes whose expression is linked to adult disease (diabetes, cancer). In fish nutrition, to our knowledge only three studies clearly explored the concept of early nutritional programming. These aimed to induce persistent metabolic adaptations, advantageous for dealing with high levels of carbohydrates [24] and low levels of long-chain polyunsaturated fatty acids [25], [26], both typical of plant-based feed. Juvenile (10 g) rainbow trout when subjected at early life (200 mg) to a short (3-day) hyperglucidic feeding period were found to display upregulated α-amylase and maltase gene expression [24]. Two other studies reported enhanced delta 6-desaturase mRNA levels in juvenile European seabass, only when they had been exposed before, at larval stage, to a dietary deficiency in long-chain n-3 polyunsaturated fatty acids [25], [26]. The adaptive responses at the molecular level were however not associated with noticeable changes in growth when fish were challenged to eat a feed rich in carbohydrates [24] or low in long-chain n-3 polyunsaturated fatty acids [25], [26]. The strong positive phenotypic response found in the present study is encouraging as a potential strategy to improve the use of plant-based diets in fish. Yet, further work needs to determine which mechanisms mediated the positive effects set forth by the early life exposure to diet V. A possible mechanism by which an organism can produce different phenotypes from a single genome in response to early life events is through altered epigenetic regulation of genes [19], [21], [22], [61], [62]. The great interest in the field of nutritional epigenetics is illustrated by the constantly growing list of food-components known to modulate epigenetic mechanisms, an important subset of which are plant compounds [61]. These studies undoubtedly open new perspectives in fish nutrition and other animal nutrition sectors in general.

In summary, our study shows that an early short term exposure of rainbow trout fry to a plant-based diet improves acceptance and utilization of the same diet when given at a later life stage. Progress in understanding the development of possible epigenetic pathways and interference of genetic predispositions in establishing such adaptive mechanism may contribute to strategies for improving the use of plant-based diets in farmed fish.

## Supporting Information

Figure S1 **Illustration of the experimental design.** Rainbow trout swim-up fry were fed for the first 3 weeks of exogenous feeding either with a plant-based diet (diet V) or with a diet containing fishmeal and fish oil as protein and fat source (diet M). This early feeding period is referred to as ‘nutritional history V or M’. After a 7-month common rearing period on diet M, both groups were challenged to feed the plant-based diet V during which voluntary FI, growth and nutrient utilisation were monitored (V-challenge).
(TIF)

Figure S2 **Growth (body weight, BW) of the fish during the pre-challenge phase (from first-feeding until the first day of V-challenge).** The trout fry were fed either diet M or V during the first 3 weeks of feeding (nutritional history M or V) and then all received diet M during the rest of the 7 month pre-challenge phase. A: Family C1; B: Family C2; C: Family C3. Data represent means from duplicate groups. The fish were transferred at ∼2.5 g (week 20) from 7°C to 16.5°C rearing temperature.
(TIF)
