# Supplementary figures and images for: The Positive Impact of the Early-Feeding of a Plant-Based Diet on Its Future Acceptance and Utilisation in Rainbow Trout
Source: PLoS One. 2013 Dec 27;8(12):e83162. doi: 10.1371/journal.pone.0083162 (PMC3873907; doi:10.1371/journal.pone.0083162)

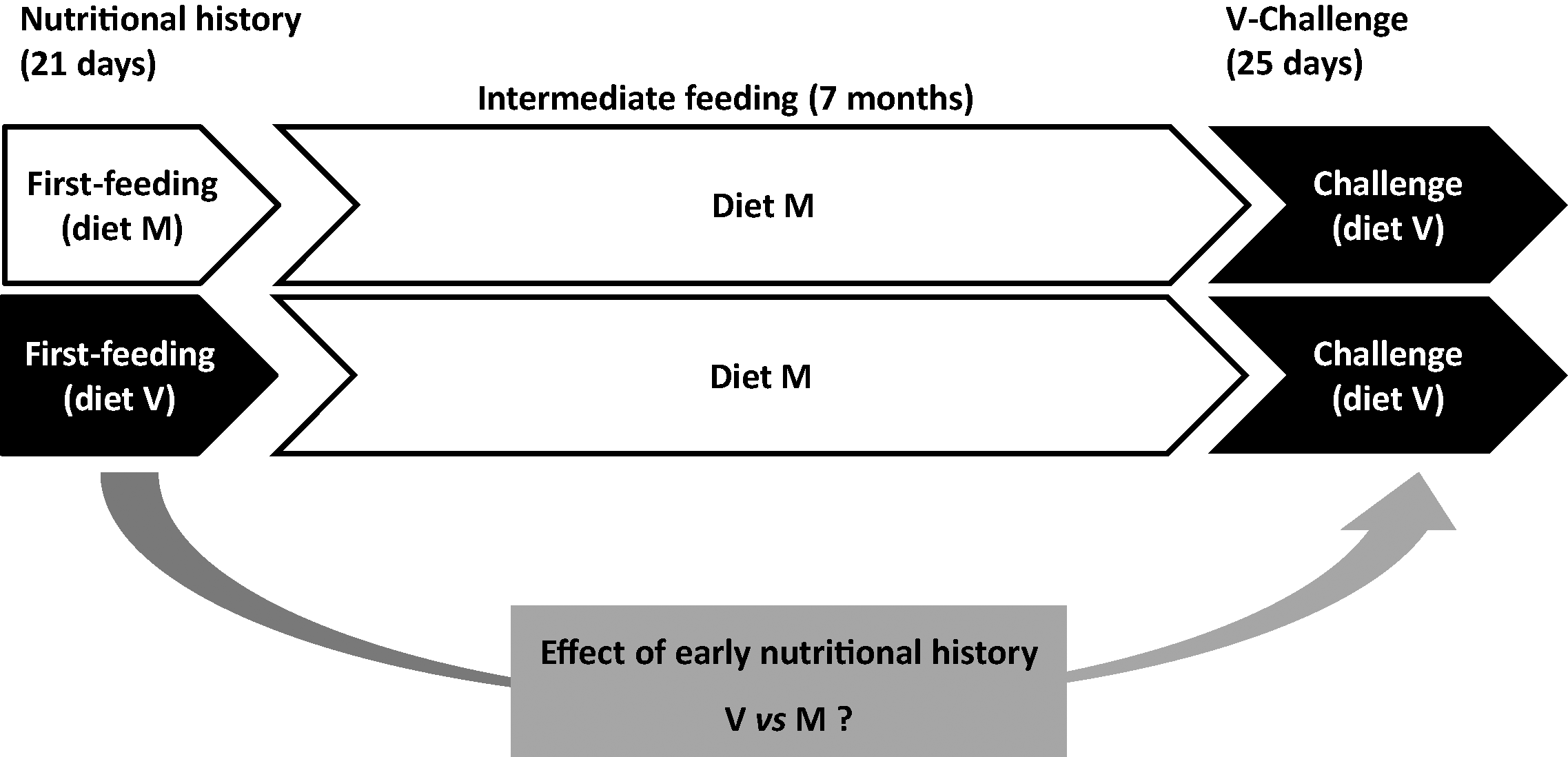

Supplement: Figure S1 — Illustration of the experimental design. Rainbow trout swim-up fry were fed for the first 3 weeks of exogenous feeding either with a plant-based diet (diet V) or with a diet containing fishmeal and fish oil as protein and fat source (diet M). This early feeding period is referred to as ‘nutritional history V or M’. After a 7-month common rearing period on diet M, both groups were challenged to feed the plant-based diet V during which voluntary FI, growth and nutrient utilisation were monitored (V-challenge). (TIF) [file pone.0083162.s001.tif]

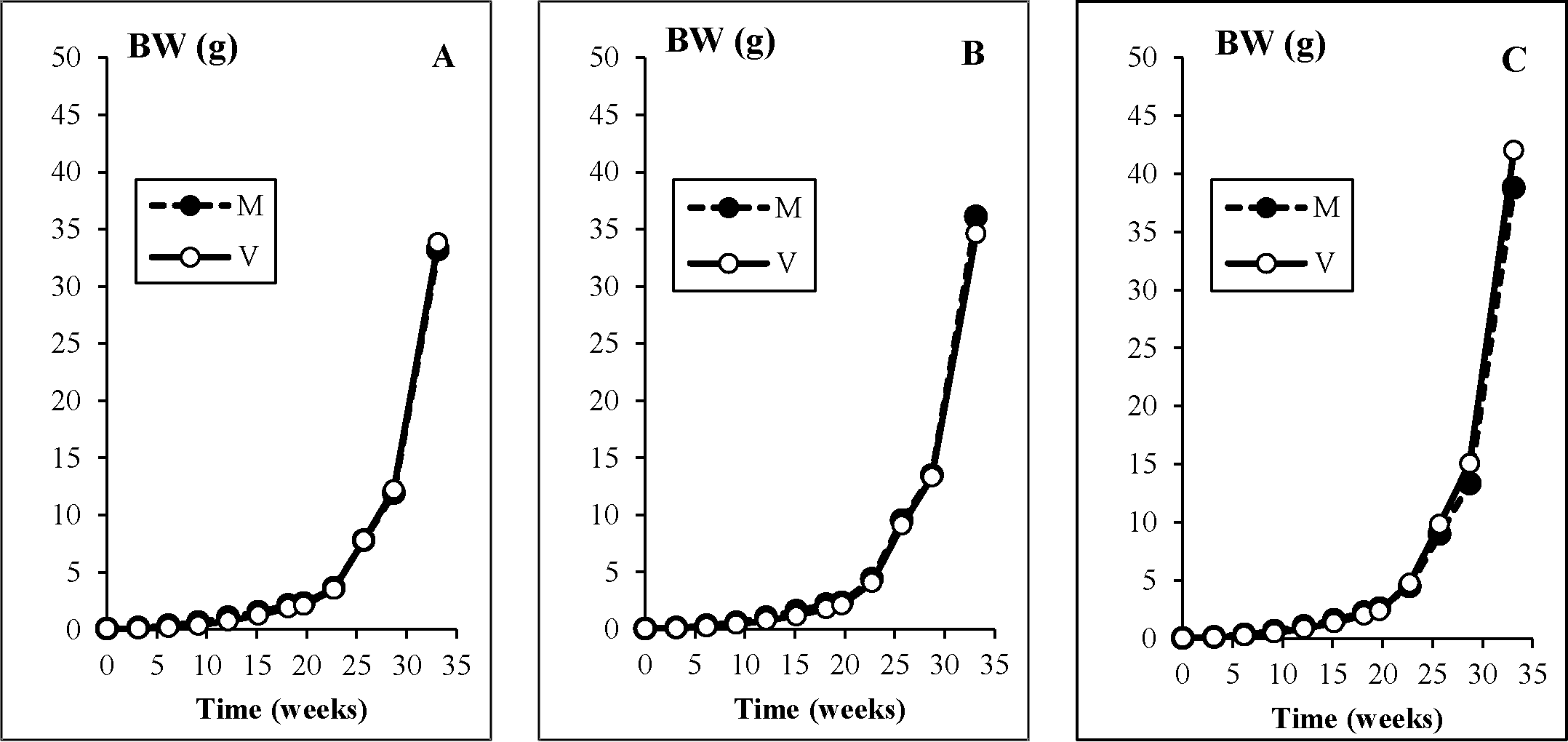

Supplement: Figure S2 — Growth (body weight, BW) of the fish during the pre-challenge phase (from first-feeding until the first day of V-challenge). The trout fry were fed either diet M or V during the first 3 weeks of feeding (nutritional history M or V) and then all received diet M during the rest of the 7 month pre-challenge phase. A: Family C1; B: Family C2; C: Family C3. Data represent means from duplicate groups. The fish were transferred at ∼2.5 g (week 20) from 7°C to 16.5°C rearing temperature. (TIF) [file pone.0083162.s002.tif]
